# Case report: A rare case of pulmonary mucormycosis caused by *Lichtheimia ramosa* in pediatric acute lymphoblastic leukemia and review of Lichtheimia infections in leukemia

**DOI:** 10.3389/fonc.2022.949910

**Published:** 2022-08-15

**Authors:** Guo-qian He, Ling Xiao, Zhen Pan, Jian-rong Wu, Dong-ni Liang, Xia Guo, Ming-yan Jiang, Ju Gao

**Affiliations:** ^1^ Key Laboratory of Birth Defects and Related Diseases of Women and Children, Ministry of Education, West China Second University Hospital, Sichuan University, Chengdu, China; ^2^ Department of Pediatrics, West China Second University Hospital, Sichuan University, Chengdu, China; ^3^ Sichuan University, Chengdu, China; ^4^ Department of Pathology, West China Second University Hospital, Sichuan University, Chengdu, China

**Keywords:** *Lichtheimia ramosa*, fungal infection, mucormycosis, acute lymphoblastic leukemia, child

## Abstract

Mucormycosis caused by *Lichtheimia ramosa* is an emerging and uncommon opportunistic infection in patients with hematological malignancies, with high mortality rates. Herein, we first report a case of pulmonary mucormycosis with *Lichtheimia ramosa* in a 3-year-old girl recently diagnosed with B-cell acute lymphoblastic leukemia. The diagnosis was made using computerized tomography of the lung, metagenomic next-generation sequencing (mNGS) of blood and sputum specimens, and microscopic examination to detect the development of *Lichtheimia ramosa* on the surgical specimen. She was effectively treated after receiving prompt treatment with amphotericin B and posaconazole, followed by aggressive surgical debridement. In our case, the fungal isolates were identified as *Lichtheimia ramosa* using mNGS, which assisted clinicians in quickly and accurately diagnosing and initiating early intensive treatment. This case also indicated the importance of strong clinical suspicion, as well as aggressive antifungal therapy combined with surgical debridement of affected tissues.

## Introduction

Mucormycosis is the third most prevalent invasive fungal disease (IFD) caused by Mucoraceae, which is widespread in the environment (1–3). It usually acts as an opportunistic pathogen, mostly in immunocompromised patients, and is aggressive with high mortality and morbidity rates (1, 4, 5). IFD is more common in patients with acute myeloid leukemia than in those with acute lymphoblastic leukemia (ALL). However, a recent study had shown that despite the recent breathtaking development of antifungal drugs, patients of ALL with prolonged neutropenia were also associated with a higher risk of acquiring IFD (6, 7).

Mucoraceae are classified into filamentous fungi. Lichtheimia ramosa, formerly known as *Absidia idahoensis*, belongs to the order of Mucorales and is currently regarded as an emerging pathogen (8–11). Mucormycosis caused by *Lichtheimia* species has increased from 5% to more than 19% in the last decade, and it is associated with dissemination and life-threatening complications (12–14). Cases have been reported in patients with diabetes, severe burns, chronic granulomatous disease, and acute myeloid leukemia (AML). The infections often develop rapidly and can potentially be angioinvasive, predominantly with pulmonary manifestations (13, 15). However, the early symptoms are unusual, and traditional etiological detection methods are ineffective. Clinically, most *Lichtheimia* infections were treated empirically with no definite diagnosis (16, 17). Early diagnosis and treatment can result in a better prognosis (4).

Here, we first report a case of lung mucormycosis in a child following induction chemotherapy for B-cell ALL. This is the first known published case of a child surviving pulmonary mucormycosis caused by *Lichtheimia ramosa* after prompt antifungal medication and aggressive surgical treatment.

## Case description

**Figure 1:**
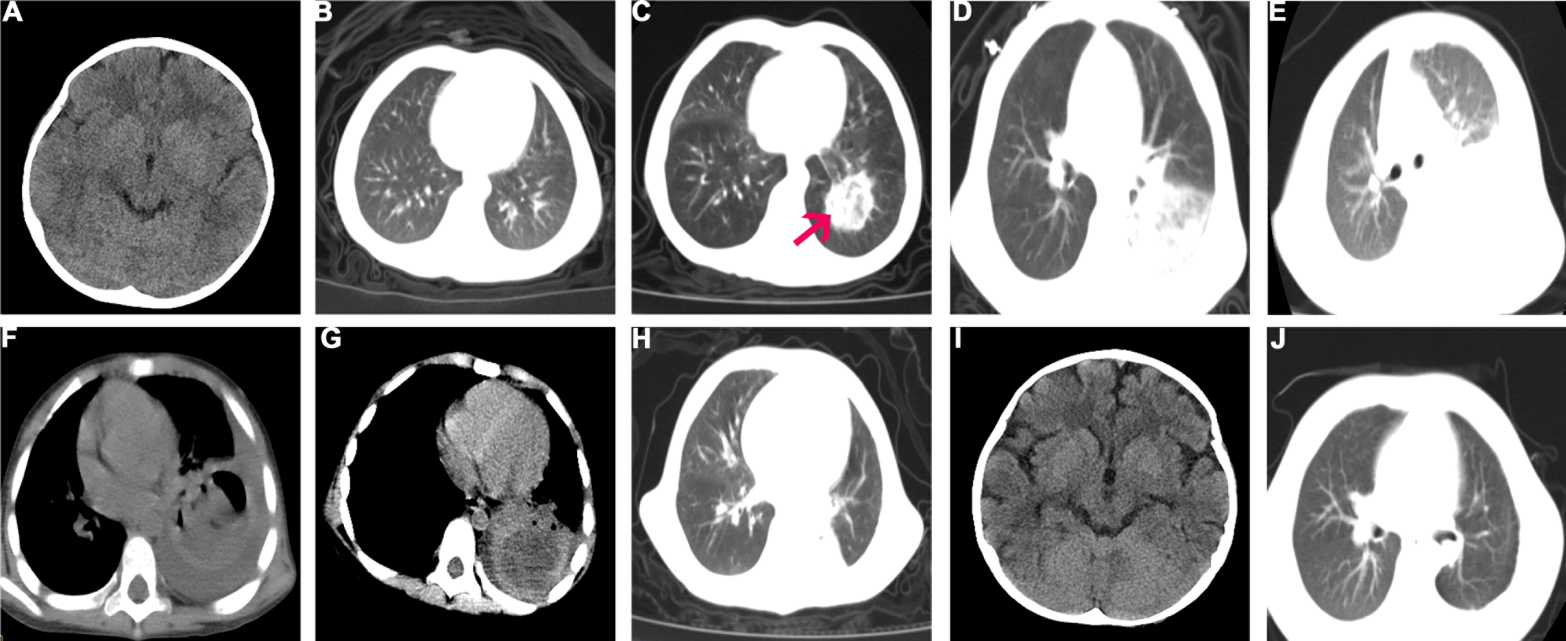
Brain and Chest CT before and after surgery. **(A)** Brain CT before chemotherapy; **(B)** Chest CT before chemotherapy; **(C)** Day 30, a nodular lesion (3.2 cm × 2.8 cm, red arrow) in the post-basal segment of the left lower lobe with ground glass opacification; **(D)** Day 38, the initial nodular lesion was larger, and new lesion in the upper lobe of the right lung; **(E, F)**. Day 52, the initial nodular lesion was 4.7 cm × 24.3 cm with part consolidation and the balloon cavity; the initial regional hazy shadow was spread diffusely in the left lung. **(G)** No regression of lesions and fungal flora in blood vessel on CTA **(I)** Brain CT on 6 months after surgery; **(J)** Chest CT on 6 months after lung lobectomy, no progression of mucormycosis.

A 3-year-old girl was admitted to West China Second University Hospital who presented with fever and a pale appearance and diagnosed with early precursor B-cell ALL with ETV6-RUNX1 fusion after MICM (morphology, immunology, cytogenetics, and molecular biology) classification. No chromosomal abnormality was detected. Before chemotherapy, the brain and chest computed tomography (CT) scan showed a negative result (**Figures 1A, B**). The patient was stratified into the low-risk group and successfully treated with induction therapy using the Chinese Children Cancer Group 2020 (CCCG-ALL-2020) protocol. It consisted of dexamethasone (6 mg/m^2^ per day, 4 days), prednisone (45 mg/m^2^ per day, 24 days), vincristine, daunorubicin, pegaspargase, and intrathecal injection of cytarabine, methotrexate (MTX), and dexamethasone (triple IT) in our hospital. The bone marrow response on day 19 (calculated from the start of chemotherapy) was evaluated using bone marrow aspiration and minimal residual disease (MRD). The bone marrow smear suggested complete remission, and the MRD was negative (<0.01%). ETV6-RUNX1 fusion gene was negative.

**Figure 2:**
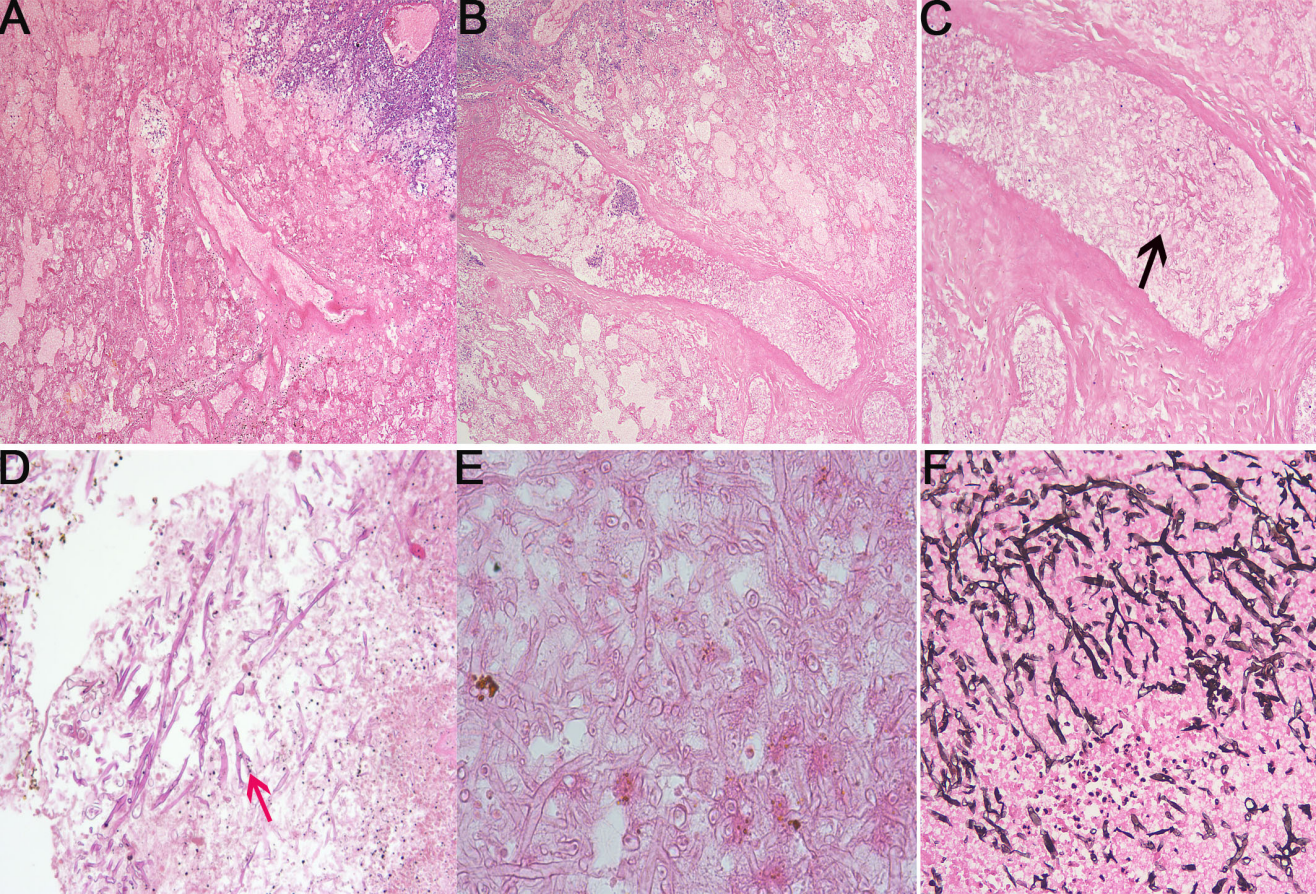
Microscopic examination of the removed lung. **(A-D)**. The tissue specimens were stained with hematoxylin and eosin staining. **(A)** Tissue necrosis *(×40).* **(B)** Tissue necrosis *(×200)*. **(C)** Fungal cenobium (black arrow) in the pulmonary alveoli (*×200*). **(D)** Filamentous fungi (red arrow) in the pulmonary (*×100*). **(E)** Filamentous fungi stained by PAS *(×200)*. **(F)** Gomori methenamine silver stain of a fungal cenobium (Grocott, ×200).

**Figure 3:**
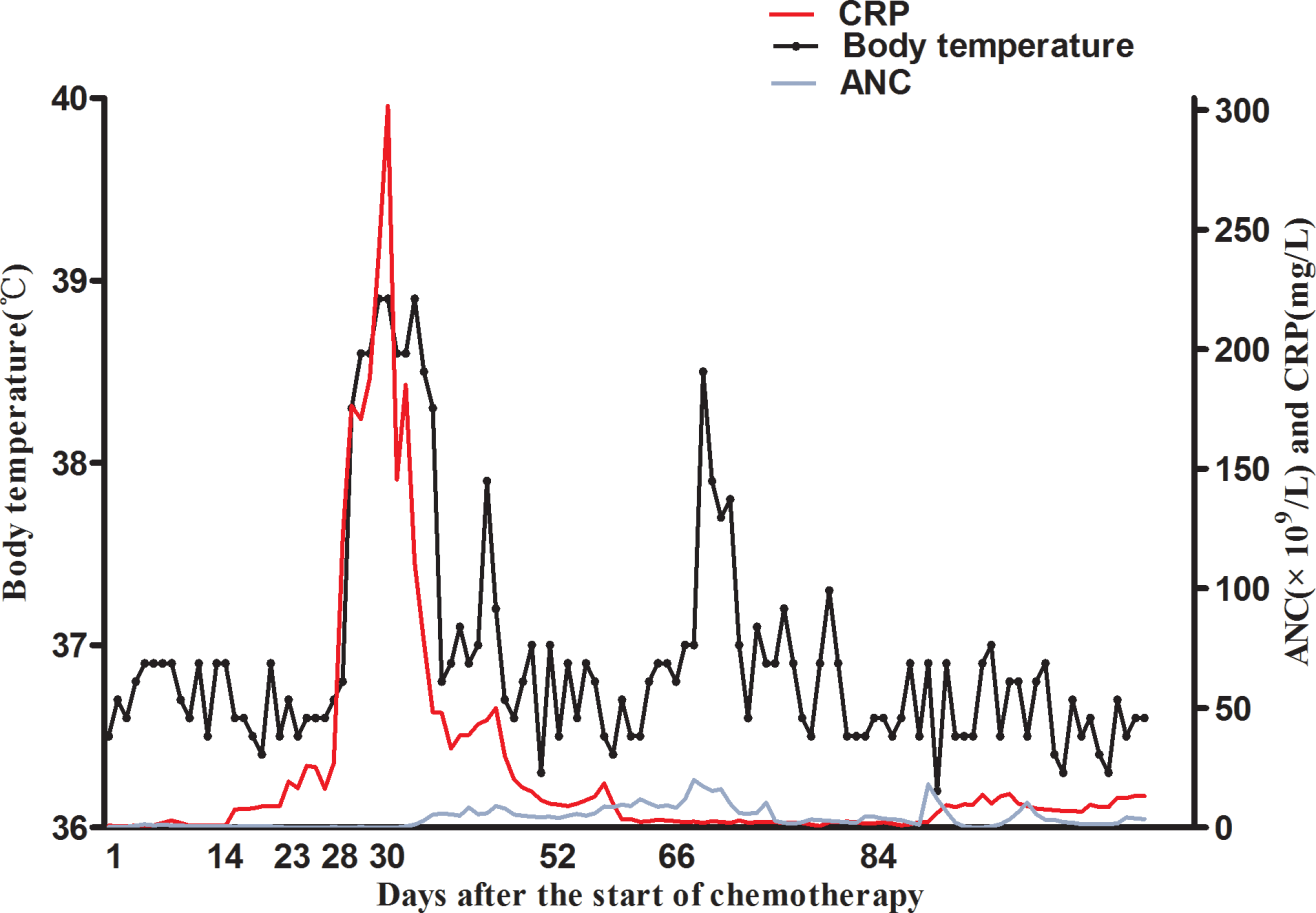
The clinical course of the patient. The patient had fever on day 28 and persistent fever for several days. Fever subsided after amphotericin B and posaconazole treatment. The ANC and CRP data were also obtained. The temperature was gradually increased on day 23 and peaked on day 34. A blood count revealed persistence of neutropenia and severe neutropenia on day 30. On day 66, surgical management was performed. On day 84, the patient started the second induction therapy.

During the first course of induction chemotherapy, the patient experienced significant neutropenia for 21 days. On day 1, piperacillin sodium and tazobactam sodium were given as empiric antibiotics, and on day 14, micafungin (4 mg/kg daily, intravenously) was added as prophylaxis for antifungal therapy if neutropenia persisted. On day 23, she complained of oral pain. On presentation, her neutrophil count was 0.02 × 10^9^/l with raised C-reactive protein (CRP) of 19.2 mg/l. Due to positive oral secretion cultures for *Burkholderia* polyphagy, the antibiotic therapy was switched to Tienam. On day 28, she complained of a fever (38.3°C), followed by a cough, dyspnea, and swelling of the face on day 30. Chemotherapy was discontinued. Results of the blood culture, serum galactomannan antigen (GM) test, and 1,3-β-D glucan test (G test) were negative. The patient was positive for rhinovirus in the throat swab and gram-positive coccus in the sputum smear. A blood count indicated that the patient had severe neutropenia (<0.05 × 10^9^/l). Procalcitonin and serum CRP levels were 1.73 ng/ml and 176.3 mg/l, respectively. Chest CT showed a nodular lesion (3.2 cm × 2.8 cm) in the post-basal segment of the left lower lobe with ground glass opacification (**Figure 1C**). The CT scans of the abdomen, as well as the cerebrospinal fluid test, all showed normal results. However, the parents later refused for the patient to undergo head CT examination due to economic reasons. Given the possibility of an opportunistic pathogen infiltrating the fungi prophylaxis and causing pulmonary infection, the antifungal therapy was changed to voriconazole, added with vancomycin. However, the high fever and dyspnea persisted. On day 34, the CRP increased to 301.8 mg/l, and following positive evolution with metagenomic next-generation sequencing (mNGS) (identified by Hugo Biotech, China, free testing) in the genomic DNA of the blood and sputum specimen, *Lichtheimia ramosa* was detected. Meanwhile, mNGS identified gram-positive streptococcus in the sputum specimen but no pathogenic prokaryotic microorganisms or viruses in the blood specimen. mNGS in cerebrospinal fluid specimens was also performed to determine intracranial IFD, although the results were negative. Because the test results were consistent with the clinical manifestations, we adjusted the antifungal treatment regimen to amphotericin B (amphotericin B liposomes were not available) intravenously (the initial dose was 0.2 mg/kg and increased up to 1.5 mg/kg gradually within 4 days); the therapeutic dose was administered intravenously for 9 weeks. The antibiotic therapy was switched to linezolid combined with Tienam. Meanwhile, the renal function was monitored and found to be normal. She experienced transient hypokalemia (2.33 mmol/l, normal range 3.5–5.5 mmol/l), which was resolved with oral and intravenous potassium supplementation. On day 38, repeated CT showed a larger initial nodular lesion and a new lesion in the upper lobe of the right lung (**Figure 1D**). On day 44, the patient’s temperature was back to normal. On day 52, a repeat CT revealed that the initial nodular lesion had gotten larger (4.7 cm × 24.3 cm) with part consolidation and that the initial regional hazy shadow had disseminated diffusely in the left lung. New regional shadows were seen in the upper lobe of the right lung (**Figures 1E, F**). Computer tomography angiography (CTA) was conducted and demonstrated no fungal flora in the blood vessels (**Figure 1G**). A multidisciplinary team (MDT) met the recommended surgical treatment for the patient. Meanwhile, posaconazole was added (6 mg/kg oral q6h) combined with amphotericin B. The blood concentration of posaconazole was in the normal range. On day 66, surgical management involved the lower lobe of the left lung and pleura (**Figure 1H**). The yellow and white fungal lesions in the basal segment of the lower lobe of the left lung were observed during surgery. Irregular fungal balls (3.0 cm) and necrosis lesions were observed in the pulmonary alveoli. A microscopic examination revealed tissue necrosis (**Figures 2A, B**), fungal cenobium in the pulmonary alveoli (**Figure 2C**), and filamentous fungi in the removed pulmonary specimen (**Figure 2D**). It was positive for periodic acid Schiff (PAS) stain **(** **Figure 2E**) and Gomori methenamine silver stain (**Figure 2F**) but negative for acid-fast stain. On day 84, the patient started the second induction therapy with cyclophosphamide cytarabine and mercaptopurine. The chemotherapy was completed without the recurrence of the fungal infection. After every 3 months of follow-up, the patient was sequentially treated with posaconazole and was continuously in complete remission. There was no progression of mucormycosis in the chest CT (**Figure 1J**) on 6 months after surgery. Brain CT was conducted after active persuasion, and it showed a good image (**Figure 1I**). The clinical course and timeline of the treatment course are summarized in **Figure 3** and **Table 1**.

**Table 1:** Timeline of events.

	Clinical course	Clinical features	Imaging results	Biology results	Antimicrobial therapy	Drug and dose
D0	Preparation of chemotherapy	Neutropenia	Normal brain and chest CT	Positive of ETV6-RUNX1 fusion	Empiric antibiotic therapy	Piperacillin sodium and tazobactam sodium 112.5mg/kg.dose, q8h intravenously
D1	Start of chemotherapy (Dex)	No fever		Normal WBC Normal CRP	Prophylaxis for PCP	Sulfamethoxazole Tablets 50mg/kg/day, twice a week, intravenously
D5	Continued of chemotherapy (Pred)	Raised of ANC		Normal WBC and CRP		
D14		Persistence of neutropenia			Adding antifungal therapy	Micafungin 50 mg/day intravenously
D19		Negative of bone marrow MRD		Negative of ETV6-RUNX1 fusion		
D23		Oral pain Raised of CRP Agranulocytosis		Positive oral secretion cultures for *Burkholderia* polyphagy	Switch anti-biotherapy	Tienam 30 mg/kg.dose, q8h intravenously
D28	Discontinued of chemotherapy	Febrile neutropenia Positive for rhinovirus and gram positive coccus		Rising of CRP and PCT Negative of Blood culture, GM,G test	Adding anti-biotherapy	Vancomycin 10mg/kg.dose q8h intravenously
D30		Persistence of fever, cough, dyspnea, and face swelling	Abnormal chest CT scan	Negative of CSF test	Switch antifungal therapy	Voriconazole 4 mg/kg.dose q12h intravenously
D34		High level of CRP		Positive of mNGS in blood and sputum: *Lichtheimia ramosa*; Negative of mNGS in CSF	Switch anti-biotherapy combined with Tienam Switch antifungal therapy	Linezolid 10 mg/kg.dose q8h intravenously Amphotericin B 0.2 mg/kg/day→1.5 mg/kg/day gradually with 4 days, intravenously
D35	Supplementation of potassium	Transient hypokalemia	Larger of lesions on image on D38	Normal of renal function		
D44		Temperature back to normal			Withdraw linezolid	
D52	Repeat CT on D52 CTA on D65 MDT meeting	Surgery recommended	More larger of lesions on CT No regression of lesions and fungal flora in blood vessel on CTA	Decreased of CRP	Antifungal combined therapy Switch antifungal therapy	Posaconazole 6mg/kg.dose q6h,oral Piperacillin sodium and tazobactam sodium 112.5mg/kg.dose, q8h, intravenously
D66	Surgery	Fungal cenobium and filamentous fungi found in the lung		Positive for PAS and Gomori methenamine silver stain		
D84	Started the second induction therapy		Regression of lesions on image			
D104	Leukemia relapse		Regression of lesions on image		Withdraw amphotericin B	

WBC, white blood cell counts; CRP, C-reactive protein; CT, computed tomography; Dex, dexamethasone (6 mg/m^2^ per day, 4 days); Pred, prednisone (45 mg/m^2^ per day, 24 days); MRD, minimal residual disease; PCP, pneumocystis carinii pneumonia; ANC, absolute neutrophil count; BM, bone marrow; GM, galactomannan antigen test; G test, 1,3-β-D glucan test; PCT, procalcitonin; CSF, cerebrospinal fluid; mNGS, metagenomic next-generation sequencing; MDT, multidisciplinary team; CTA, computer tomography angiography; PAS, periodic acid Schiff.

## Discussion and review of the literature

Mucormycosis is an opportunistic invasive fungal infection caused by Mucoralean and zygomycetes fungus, like the *Lichtheimia* species. This fungus is found almost ubiquitously in soil, farming products, and processed and unprocessed food products (12, 18). Despite being classified as a low-virulence pathogen, the percentage of Mucor infections caused by *Lichtheimia* species has increased from 5% to more than 19% in the last decade, according to recent reviews (13, 19). *Lichtheimia ramosa* is the most common pathogenic *Lichtheimia* species and a leading cause of mucormycosis (15). The clinical disease resembles infections with other mucoralean fungi. The pulmonary is the most common organ infected by *Lichtheimia ramosa*. Cutaneous and subcutaneous were also observed in some cases (18). Most patients with serious mucormycosis underlying diseases, such as hematological malignancies, are predominantly at risk (1). Classically, the risk of mucormycosis has been considered higher in AML and HSCT than in ALL (20–22). In this study, reports about *Lichtheimia ramosa* mucormycosis in leukemia published in literature were reviewed. Here, we performed a detailed review of *Lichtheimia ramosa*-induced mucormycosis events in leukemia based on available case reports (**Table 2**). For the cases reviewed, there have been rare reports of *Lichtheimia ramosa* infection in leukemia (23–25). The three cases were all reported with AML, with one case including an adult patient’s skin mucormycosis. The other child with AML had pulmonary and cerebral mucormycosis caused by *Lichtheimia ramosa*. However, the episode was rare in ALL for the patient infected with *Lichtheimia ramosa*. This is the first reported case of pediatric ALL in our study. Risk factors are indeed a matter of great concern to us. A thorough search for risk factors can aid in the reduction of opportunistic infections and improve patient prognosis. In the case of our patient, it would be reasonable to suppose that the infection occurrence was related to immunocompromised status induced by hematological malignancies; the ETV6-RUNX1 gene is a marker of good prognosis for ALL. However, clinical studies have shown that ALL patients carrying this gene are more susceptible to severe opportunistic infections, which may be associated with high susceptibility to chemotherapy, resulting in long-term severe granulocytosis. For this patient, in addition to the above risk factors, living near garbage sites, vegetable markets, and flower shops as well as poor sanitation may increase the risk of fungal infection. At the same time, long-term poor appetite and poor nutritional status may increase the risk of infection due to malnutrition and primary or secondary endocrine disorders, such as diabetes. Normal blood glucose levels were monitored, as was her nutrition level. During the diagnosis and treatment of the patient, blood glucose and urine glucose levels were monitored regularly. Strengthening nutritional support is beneficial for improving the prognosis.

**Table 2:** *Lichtheimia ramosa*-related mucormycosis based on available case reports.

	David Navarro	Cateau	Suzuki
Age (years)	10	27	8
Sex	Female	Male	Male
Baseline diagnosis	AML	AML	AML
Relapse	Second relapse	No	First relapse
Chromosomal or genetic abnormalities	Data missing	46 XY [23]/47 XY, ?4 [10]	No
Persistent febrile neutropenia	Yes	Yes	Yes
Antifungal or anti-mold prophylaxis	Yes	Yes	Yes
Presence of symptoms Fever Cough Others	Yes Yes Dyspnea	Yes No Chills	No No Right chest pain Bloody sputum Nephritis-like symptoms
Type of infection site	Pulmonary	Skin	Pulmonary Cerebral
GM/G test	Positive (GM)	Data missing	Negative (G test)
Proven mold infection/specimen	BAL fluid	necrosis samples culture	Sputum culture
Treatment for mycosis	Amphotericin B Posaconazole	Liposomal amphotericin B posaconazole	Liposomal amphotericin B Stem cell transplantation
Surgery	No	No	No
Outcome	Death	Death	Death

Early detection is critical for avoiding the aggressive clinical course and improving prognosis. The diagnosis is based on a combination of clinical examinations and histopathologic and radiological investigations. A biopsy and cultures on standard mycological media are typically required for a definitive diagnosis (26, 27). With the progression of the disease, persistent fever and respiratory system symptom may occur. While the early symptoms are not typical, they cannot be distinguished from other infective causes from other pathogens. Although CT data may be suggestive, they are frequently non-specific (27–29). As recommended by the ECIL guidelines, we also wanted to screen for intracranial infection, including the possibility of endocardial infection. In fact, we recommend that cranial imaging be as complete as possible, even if the patient does not have neurological signs and symptoms. Additionally, proper tissue sampling was performed mostly after a delay for the fungal detection. Microscopically, rhizoids are rarely observed on *Lichtheimia* strains. Bronchoalveolar lavage fluid (BAL fluid) and sputum cultures are clinically much feasible and safer than tissue biopsy (13, 30). Diagnosis of mucormycosis is often missed or delayed. In our case, the parents refused BALF due to risk. It is difficult to detect *Lichtheimia ramosa* using traditional etiological detection methods. Definitive identification requires molecular methods. mNGS is a rapid and non-invasive diagnostic method. Using mNGS as soon as possible is recommended when an infection with a rare pathogen is suspected, especially in immunocompromised individuals who need emergency treatment (31, 32). Early clinical trials of patients with meningitis or encephalitis, invasive fungal infections, community-acquired pneumonia, and other clinical indications revealed the potential of direct-from-specimen mNGS in enabling a difficult infection diagnosis. However, in published studies, the proportion of patient cases having a positive clinical impact as a result of mNGS testing is low, and the expense of testing is high, emphasizing the importance of improving our understanding of “when to test” and for which patients mNGS testing is appropriate (33–36). In our case, we used mNGS for detecting this rare species. It enabled us to effectively treat this child patient with lung mycosis caused by a *Lichtheimia ramosa* infection.

According to our literature review, the empiric antifungal treatment of *Lichtheimia ramosa* mucormycosis is amphotericin B and posaconazole. Lichtheimia is usually sensitive to amphotericin B and posaconazole. Amphotericin B is the most active drug against *Lichtheimia* species and is the only antifungal agent recommended in pediatric mucormycosis (37–39). Liposomal amphotericin B (LAmB) is a standard of care for a wide range of medically important opportunistic fungal pathogens. LAmB has a significantly improved toxicity profile compared with conventional amphotericin B. However, the adverse effects and especially nephrotoxicity of amphotericin B, even with the liposomal formulation, could be problematic. Moreover, amphotericin B liposomes were not available in the region of China, so we had to choose conventional amphotericin B and tried to exclude the intracranial fungal infection. However, it also has many side effects, which even causes the suspension of antifungal therapy. According to our experience, it usually causes severe vomiting and electrolyte disorder to start with the therapeutic dose on day 1, so we tried to increase the dose gradually. As we hoped, the intolerance of the patient was good. Her renal function was persistently normal, and she suffered transient mild hypokalemia only.

Posaconazole, possibly the recommended oral therapy, can be used to complement or substitute amphotericin B therapy (3, 40–42). However, there is no intravenous therapy, and it is not easy to monitor the blood drug concentration. The effectiveness of posaconazole is still controversial (43). It has been reported that voriconazole has no activity against *Lichtheimia ramosa*. Isavuconazole has been approved for adults as first-line therapy if amphotericin B treatment is not appropriate (21, 44). Isavuconazole has rarely been reported to be used in children with mucormycosis (45). As recommended by the guidelines, appropriate use of antifungal agents includes dosage, route of administration, and monitoring of blood concentration. We do want to monitor posaconazole blood concentrations, and we want to use an intravenous preparation when amphotericin B is combined with posaconazole during the patient’s hospital stay. However, due to the limited conditions, posaconazole intravenous preparations could not be obtained at that time, nor could blood drug concentration monitoring TDM be done. That is something we need to work on. It is also recommended that blood concentrations (effective therapeutic concentration) be monitored as much as possible. It has been reported that echinocandin (micafungin and caspofungin) in combination with amphotericin B also has a certain effect (42). Mucormycosis is treated with a combination of antifungal medications and surgical debridement of affected tissues. Surgery has been demonstrated to improve patient survival (43). Moreover, a combination strategy of intravenous inhalation and local inhalation and direct airway perfusion of amphotericin B may be an effective strategy for the treatment of pulmonary mucormycosis (46, 47). In our case, intravenous liposomal amphotericin B was started just after appearance of suspicious clinical presentation and radiological findings supporting mucormycosis. The patient was then given posaconazole in conjunction with amphotericin B, as well as prompt surgical debridement. *Lichtheimia ramosa* has characteristics comparable to invasive aspergillosis, including the potential for angioinvasion and dissemination (1, 48). Extensive angioinvasion results in tissue necrosis (49). Pulmonary hemorrhage, hemoptysis, asphyxia, and intracranial infection should be watched out for during diagnosis and treatment. Fortunately, the patient had no pulmonary hemorrhage and no abnormal cranial imaging and CSF findings. If IFD is diagnosed, antifungal treatment should be started immediately, and chemotherapy should be postponed until the IFD is under control, except in rare cases needing prompt anti-leukemic intervention. During the treatment, renal work and electrolyte (low potassium) were closely monitored (24). In this case, renal function was normal. She had transient hypokalemia, improved by oral and intravenous potassium supplementation and improved diet. In addition, the prompt recovery of chemotherapy-induced neutropenia is also necessary. Fortunately, the child in this case had low-risk ALL with sustained CR in BM after experiencing induction remission therapy. Since the risk factors for infection in this patient are of concern, ALL with *Lichtheimia ramosa* infection is extremely rare. The patient received amphotericin B monotherapy for 5 weeks, amphotericin B+ posaconazole for 4 weeks, and then posaconazole monotherapy. The specific doses are shown in the attached table. She then had entered the maintain chemotherapy of the CCCG-ALL-2020 therapy for the low-risk group and stop using antifungal drugs. During long-term follow-up, blood routine test, CRP test, and CT were taken. The head and chest CT scans were all normal. The parents and children had good compliance and tolerance.

## Conclusion

Here, we report a rare case of an infrequent fungal mycosis associated with the causal species (*Lichtheimia ramosa*) in a child ALL and review *Lichtheimia ramosa*-related mycosis based on published case reports. This report summarizes the difficulty of making an early diagnosis and the importance of considering mucormycosis in subjects developing fever unresponsive to other antimicrobials and have breathing difficulty. The timing of proper medical therapy with antifungal medications and early surgical resection is critical. mNGS is more effective when used early in the diagnostic process.

## Data availability satement

The original contributions presented in the study are included in the article/supplementary material. Further inquiries can be directed to the corresponding author.

## Ethics statement

The studies involving human participants were reviewed and approved by the Center for Ethics in the West China Second University Hospital of Sichuan University. Written informed consent was obtained from the minor(s)’ legal guardian/next of kin for the publication of any potentially identifiable images or data included in this article.

## Author contributions

GH: analyzed the patient data and drafted the manuscript. LX: provided significant contributions to the interpretation of CT and CTA imaging. ZP: made significant contributions to the collection of patient data. DL provided significant contributions to the analysis of pathological data. JW and XG provided significant contributions to the analysis of the patient data. MJ: designed the case report and revised the manuscript. JG: writing–review and editing. All authors contributed to the article and approved the submitted version.

## Funding

This study was funded by the Sichuan Science and Technology Program, grant no. 2021YFSY0040-LH and 2022YFS0236 (to GQH), and the Universal Application Project of Sichuan Provincial Health and Family Planning Commission of China, grant no. 18PJ032(to GQH).

## Acknowledgments

Wei Jiang and Juan Zou provided selfless assistance with the analysis of pathological data.

## Conflict of interest

The authors declare that the research was conducted in the absence of any commercial or financial relationships that could be construed as a potential conflict of interest.

## Publisher’s note

All claims expressed in this article are solely those of the authors and do not necessarily represent those of their affiliated organizations, or those of the publisher, the editors and the reviewers. Any product that may be evaluated in this article, or claim that may be made by its manufacturer, is not guaranteed or endorsed by the publisher.
